# Leaf morphology, optical characteristics and phytochemical traits of butterhead lettuce affected by increasing the far-red photon flux

**DOI:** 10.3389/fpls.2023.1129335

**Published:** 2023-08-02

**Authors:** Ellen Van de Velde, Kathy Steppe, Marie-Christine Van Labeke

**Affiliations:** ^1^ Horticultural Sciences, Department of Plants and Crops, Faculty of Bioscience Engineering, Ghent University, Ghent, Belgium; ^2^ Laboratory of Plant Ecology, Department of Plants and Crops, Faculty of Bioscience Engineering, Ghent University, Ghent, Belgium

**Keywords:** lettuce, far-red photon flux, light-emitting diodes, leaf morphology, leaf reflectance, light use efficiency, indoor farming, proximal sensing

## Abstract

Light and its spectral characteristics are crucial for plant growth and development. The far-red photon flux mediates many plant processes through the action of phytochrome and also accelerates the photosynthetic electron transfer rate. In this study, we assessed the effects of far-red addition on butterhead lettuce morphology, light use efficiency, optical properties, and phytochemical characteristics. Three-week-old lettuce plants (*Lactuca sativa* L. cv. Alyssa) were grown for up to 28 days under a 10% blue and 90% red light spectrum (200 µmol m^-2^ s^-1^, 16 h photoperiod) to which five different intensities of far-red light (peak at 735 nm) were added (0-9-18-36-72 µmol m^-2^ s^-1^). White light-emitting diodes were included as a proxy for sunlight. Increasing supplemental far-red photon flux from zero to 21% increased the light use efficiency (g per mol) by 37% on day 14; 43% on day 21; and 39% on day 28. Measurements of projected head area suggest that this was associated with an increase in leaf expansion and photon capture and not necessarily a direct effect on photosynthesis. Moreover, vegetation indices based on leaf reflectance showed a decrease in chlorophyll-related indices under a high far-red photon flux. This decrease in pigment content was confirmed by chemical analyses, suggesting that the plants may not reach their full potential in terms of photon capture, limiting the overall photosynthetic performance. Furthermore, the stress-related Carter 1 index increased in plants grown under a high far-red photon flux, indicating early plant stress. Far-red tended to decrease the content of total phenolics and increase soluble sugars. The higher sugar levels can be attributed to an improved photochemical efficiency due to photosystem I excitation by far-red wavelengths, also known as the Emerson Enhancement effect. Despite these higher sugar levels, no effect on foliar nitrate content was observed. Our results show that far-red supplementation has the potential to enhance light interception at the early growth stages, although higher intensities of far-red may cause plant stress.

## 1 Introduction

In recent years, the concept of vertical farming gained attention as a way of increasing horticultural productivity, supporting the sustainable cultivation of high-quality food products (Van Gerrewey et al., 2022). It offers the opportunity to obtain a higher and more predictable yield per unit area, meanwhile optimizing the use of water and pesticides (van Delden et al., 2021). Moreover, as horticultural production is largely decoupled from land use, these production facilities can be located near cities, reducing food miles while assuring food security (Eigenbrod and Gruda, 2015). Vertical farming can take place either in greenhouses or in fully-enclosed plant factories. Greenhouses provide the benefit of free sunlight as a main light source, although the lower areas of multilayer systems are often shaded by higher levels of planting. In closed plant factories, sunlight is absent altogether (Beacham et al., 2019). Therefore, these facilities are largely dependent on artificial lighting, characterized by its light intensity and spectral composition. In the last few years, a lot of research has been conducted on optimizing the red/blue ratio for leaf lettuce production (Wang et al., 2016; Pennisi et al., 2019; Spalholz et al., 2020). As recent horticultural light emitting diode (LED) fixtures are often able to emit far-red photons, it is pivotal to characterize the effects of far-red photons on plant growth and morphology, especially since there are indications that including far-red in the growth spectrum may also improve water use efficiency in vertical farms (Carotti et al., 2023).

Traditionally, photosynthetically active radiation (PAR) is defined as the range between 400 and 700 nm, comprising blue (400-500 nm), green (500-600 nm) and red (600-700 nm) photons (McCree, 1972). Recently, it was suggested that far-red photons (700-750 nm) should be included in the definition of photosynthetic photons. These photons are minimally absorbed by leaves and have negligible photosynthetic activity when applied alone, but show synergistic effects when applied together with photons in the 400-700 nm range (Zhen et al., 2021). This enhancement effect was already observed by Emerson et al. (1957), but until recently, its implications for crop production received little attention. It has been shown by Zhen and van Iersel (2017) that the addition of 110 µmol m^-2^ s^-1^ of far-red (700-770 nm) to an increasing intensity of red and blue or white light (ranging from 50 to 750 µmol m^-2^ s^-1^) increases the efficiency of photosystem II in lettuce. Far-red light preferentially excites photosystem I, which tends to be under-excited relative to PSII in absence of far-red, limiting the overall photosynthesis efficiency. By supplementing far-red to red and blue or white light, the excitation balance between the two photosystems may be restored, increasing photochemical efficiency and thus carbohydrate synthesis (Zhen and van Iersel, 2017; Zhen and Bugbee, 2020). Zhen and Bugbee (2020) reported that far-red photons (700-750 nm, peak at 735 nm) supplemented to cool white light were equally effective as photons in the 400-700 nm range, provided that the share of far-red photons did not exceed about 30% of the total photon flux from 400 to 750 nm. They also found that, when adding far-red LEDs with peak wavelengths of 711, 723 and 746 nm to a red/blue spectrum, the increase in photosynthesis decreased with increasing peak length. Because of these effects on photosynthesis, the wavelength range from 400 to 750 nm is recently termed extended PAR or ePAR (Zhen et al., 2021).

Besides its role in photosynthesis, the far-red photon flux plays a critical role in photomorphogenesis. Phytochromes are converted to their inactive forms (Pr) upon excitation by far-red light (peak absorption at 730 nm), whereas red radiation (peak absorption at 670 nm) converts them back to their active state (Pfr) (Sharrock, 2008). In natural light circumstances, there is a dynamic balance between these forms, which is largely determined by the far-red fraction of the ambient light (Shinomura et al., 2000). The far-red photon flux can thus modulate plant responses to red light. When light is transmitted through a leaf, the transmitted light is enriched in far-red photons as these are less absorbed by the leaves. Therefore, plants grown under higher levels of planting or shaded by neighboring plants will be exposed to higher far-red fractions. A high far-red fraction elicits shade-avoidance responses, such as faster elongation of stems and leaves (Franklin, 2008). When far-red is supplemented to the spectrum, plants often show an increased total leaf area, a higher biomass production and a higher specific leaf area (SLA), indicating that the leaves are thinner when grown under higher far-red fractions. For example, Mickens et al. (2018) compared white LEDs (20% blue, 48% green, 32% red, total PAR flux 180 µmol m^-2^ s^-1^) to white LEDs with the same spectrum and intensity, but supplemented with 34 µmol m^-2^ s^-1^ of far-red photons. They observed a 14.9% increase in the fresh mass of lettuce, while the leaf area increased by 27.8% and the specific leaf area increased with 19.4%. Meng and Runkle (2019) also studied the effect of far-red on lettuce and supplemented 180 µmol m^-2^ s^-1^ of red and blue LEDs (blue/red ratio 1/1) with 0, 30 and 75 µmol m^-2^ s^-1^ of far-red photons. They report that fresh biomass did not increase under supplemental far-red, while dry biomass increased by up to 24% when 75 µmol m^-2^ s^-1^ of far-red was added. Since high far-red fractions are often associated with lower PAR fluxes in natural (shaded) environments, it is important to note that the shade-avoiding responses may be the result of the interaction between PAR flux and far-red fraction. Legendre and Iersel (2021) found no interactive effects of PAR flux and far-red fraction for lettuce, while Kusuma and Bugbee (2023) report an increase in leaf area and dry mass accumulation when far-red fractions are increased at high PAR fluxes, but a decrease in leaf area and dry mass accumulation at lower PAR fluxes.

Although increasing the far-red flux may have beneficial effects on photosynthesis and biomass accumulation, this can happen at the expense of the contents of photosynthetic pigments. Lower levels of chlorophyll and carotenoids are often reported when plants are grown under far-red addition (Wong et al., 2020; Kong and Nemali, 2021). The sugar content, on the other hand, is often increased when plants are grown under higher far-red fractions (e.g. Zou et al., 2021; Zou et al., 2023). Since the sugar metabolism is closely linked with the metabolism of nitrate in lettuce (Behr and Wiebe, 1992) and because nitrate has important implications for human health (Hmelak Gorenjak and Cencič, 2013), it is necessary to assess the effects on the nitrate content. Finally, the effects on plant secondary metabolites should be investigated, since these are indicative of plant stress and also have antioxidant properties (Geilfus, 2019).

The vast majority of studies on far-red addition focused on leaf lettuce. However, plant responses to far-red photons are proven to be cultivar specific (Liu and Van Iersel, 2022). Moreover, butterhead lettuce (*Lactuca sativa* L. var. *capitata*) is economically more important in Belgium, accounting for 14.8% of the cultivation area under glass, compared to only 3.0% for other lettuce types (Platteau et al., 2018). Head formation is an important stage in head lettuce production and is characterized by a drop in the leaf length-to-width ratio below 1 (Bensink, 1971). Since far-red influences leaf expansion, the onset of head formation could be influenced by the far-red photon flux. Hence, far-red addition may impact the time necessary to obtain marketable plants. In the present study, we investigated the response of butterhead lettuce plants to different doses of far-red as a supplement to red and blue LED lighting. We hypothesized that the far-red flux would affect the morphology of the leaf blade and overall shoot weight, possibly at the expense of phytochemical contents, such as photosynthetic pigments. In addition, we hypothesized that spectral indices obtained by non-destructive proximal sensing can discriminate physiological differences caused by the applied far-red doses.

## 2 Materials and methods

### 2.1 Plant materials and experimental setup

The experiment was conducted in growth chambers of the Faculty of Bioscience Engineering (Ghent University, Belgium). The growth chambers were controlled for temperature and monitored for relative humidity. Seeds of green butterhead lettuce (*Lactuca sativa* L. cv. ‘Alyssa’, Rijk Zwaan, the Netherlands) were sown in Jiffy-7 pellets (Jiffy Products International B.V., Zwijndrecht, the Netherlands) saturated with nutrient solution. This nutrient solution contained 0.59 mM NH_4_^+^, 6.74 mM K^+^, 4.22 mM Ca^2+^, 1.69 mM Mg^2+^, 13.58 mM NO_3_^-^, 1.35 mM H_2_PO_4_^-^ and 2.11 mM SO_4_^2-^. 0.02 g/L micronutrients (Chelal Flor NF, BMS Micro-Nutrients NV, Bornem, Belgium) were added, resulting in 15.70 µM B, 0.98 µM Cu^2+^, 27.20 µM Fe^3+^, 8.01 µM Mn^2+^, 0.52 µM Mo and 3.98 µM Zn^2+^. Germination and seedling establishment took place at 18.0 ± 0.5°C under white LEDs (NS1, Valoya, Helsinki, Finland, PAR flux 185.4 ± 18.7 µmol m^-2^ s^-1^, consisting of 21.3% blue [400-500 nm], 41.5% green [500-600 nm] and 37.2% red [600-700 nm], far-red intensity 11.8 ± 1.4 µmol m^-2^ s^-1^ [700-770 nm]) under a 16h/8h light/dark regime.

After three weeks, 36 plantlets per light treatment were randomly divided over six growth containers (40 × 30 × 12 cm, L × W × H, plant density 50 plants m^-1^), containing 4 L of nutrient solution (composition as described above), put in vertical rack compartments and subjected to the light treatments described below. The treatments were separated from each other by non-transparent plastic foil. Fourteen days after transplantation (DAT), three plants from each growth unit were harvested to simulate the decrease in plant density in a moving gutter system and to prevent overlap of leaves of different plants, so the observed effects could be attributed only to the far-red doses that were applied in the light spectrum. The remaining plant density was 25 plants m^-2^. At 21 DAT, two of the remaining plants from each growth unit were collected, decreasing the plant density further to 8.3 plants m^-2^ until the final harvest at 28 DAT, after the onset of head formation. To prevent algae growth, the nutrient solution was shielded from the light by a non-transparent plastic foil. In addition, aeration in the nutrient solution was provided by aerator stones. To monitor air temperature and relative humidity, one DL-USB-173 temperature/RH datalogger (ATAL, Purmerend, the Netherlands) per treatment was placed between the growth containers, while the EC and pH of the nutrient solution were measured twice a week using a HI991301 pH/EC/TDS meter (Hanna Instruments, Temse, Belgium). If the EC dropped below 2.0 dS/m or if the pH rose above 6.5, the nutrient solution was discarded and replaced. Throughout the experiment, the EC averaged around 2.29 ± 0.18 and the pH around 6.11 ± 0.36. Air temperature and humidity data are presented in **Table 1**.

**Table 1:** Cultivation and photon flux details per light treatment.

Treatment code	Air temperature (°C)	Relative Humidity (%)	Blue photon flux [400-500 nm] (µmol m^-2^ s^-1^)	Green photon flux [500-600 nm] (µmol m^-2^ s^-1^)	Red photon flux [600-700 nm] (µmol m^-2^ s^-1^)	PAR photon flux [400-700 nm] (µmol m^-2^ s^-1^)	Far-red photon flux [700-750 nm] (µmol m^-2^ s^-1^)	ePAR photon flux [400-750 nm] (µmol m^-2^ s^-1^)	Red/far-red ratio	PSS	Far-red fraction
FR00	18.4 ± 0.0	71.1 ± 0.1	21.2 ± 0.5	0.1 ± 0.0	178.7 ± 4.3	200.0 ± 4.7	0.0 ± 0.0	200.0 ± 4.7	∞	0.886	0.00
FR09	17.6 ± 0.0	74.2 ± 0.1	22.7 ± 0.6	0.1 ± 0.0	184.7 ± 3.8	207.5 ± 4.3	8.3 ± 0.2	215.8 ± 4.5	29.29	0.874	0.04
FR18	17.1 ± 0.0	80.3 ± 0.1	22.0 ± 0.9	0.1 ± 0.0	167.0 ± 6.2	189.3 ± 7.1	15.7 ± 0.6	205.0 ± 7.7	15.61	0.863	0.07
FR36	18.3 ± 0.0	78.2 ± 0.1	19.1 ± 0.6	0.1 ± 0.0	142.5 ± 3.9	161.9 ± 4.5	25.8 ± 0.7	187.8 ± 5.2	8.12	0.844	0.12
FR72	18.2 ± 0.0	78.7 ± 0.2	22.5 ± 0.6	0.1 ± 0.0	164.1 ± 3.9	187.1 ± 4.5	58.1 ± 1.4	245.2 ± 6.0	4.27	0.809	0.21
White	18.0 ± 0.0	76.5 ± 0.1	40.9 ± 1.2	77.9 ± 2.2	77.2 ± 1.9	196.3 ± 5.3	13.0 ± 0.3	209.3 ± 5.6	4.62	0.828	0.18

Air temperature and relative humidity were monitored at one point per treatment and averaged over the growing period (mean ± SE). Photon flux data shown are means ± SE of ten measurements per treatment. The red/far-red ratio was calculated as described by Holmes and Smith (1977) by dividing the photon flux from 655 to 665 nm (red) by the photon flux between 725 and 735 nm (far-red). PSS was estimated according to Sager et al. (1988). The far-red fraction was calculated as suggested by Kusuma and Bugbee (2021).

Light treatment settings consisted of a 10% blue (peak at 460 nm) and 90% red (peak at 660 nm) spectrum under a 16h/8h light/dark regime (GreenPower LED dynamic module, Signify, Eindhoven, the Netherlands), to which five far-red (peak at 735 nm) doses (0-9-18-36-72 µmol m^-2^ s^-1^) were added. White LEDs (NS12, Valoya, Helsinki, Finland) were included as a proxy for sunlight. PAR flux settings were kept at 200 µmol m^-2^ s^-1^ for all treatments. The actual spectral light compositions were measured at plant level using a SS-110 spectroradiometer (Apogee Instruments, Logan, UT, USA) and are shown in **Table 1** and **Supplementary Figure 1,** indicating a significant deviation in PAR flux for FR36, and large deviations in far-red photon flux for FR36 and FR72 compared to the settings. To facilitate interpretation, however, the treatment codes used in this work will refer to the light settings instead of the measured values. The red/far-red ratio was calculated as described by Holmes and Smith (1977) as the photon flux between 655 and 665 nm divided by the photon flux between 725 and 735 nm. Based on Sager et al. (1988), the phytochrome photostationary state (PSS = Pfr/[Pfr+Pr]) was estimated by multiplying the measured photon flux at every wavelength with the relative absorption for each form of phytochrome:


$$\text{PSS}=\;\frac{\sum_{340}^{800}{\text{N}}_{\text{λ}}{\text{σ}}_{\text{rλ}}}{\sum_{340}^{800}{\text{N}}_{\text{λ}}{\text{σ}}_{\text{rλ}}+\;\sum_{340}^{800}{\text{N}}_{\text{λ}}{\text{σ}}_{\text{frλ}}}$$

with N_λ_ the measured photon flux at wavelength λ and σ_rλ_ and σ_frλ_ the phytochemical cross-sections of phytochrome at wavelength λ for the red and far-red absorbing state, respectively. Recently, the far-red fraction, defined as


$$\text{Far-red fraction}=\;\frac{\text{far-red photon flux}}{\text{red photon flux}+\text{far-red photon flux}}$$

was proposed as a more intuitive metric for phytochrome effects (Kusuma and Bugbee, 2021). For this calculation, we took into account the red photon flux from 650 to 670 nm and the far-red photon flux between 720 and 740 nm, as suggested by the authors. As the far-red fraction shows a positive correlation with phytochrome-mediated plant responses, this metric is more intuitive and therefore, we will mainly refer to this metric in the results and discussion sections.

### 2.2 Plant morphology

**Figure 1:**
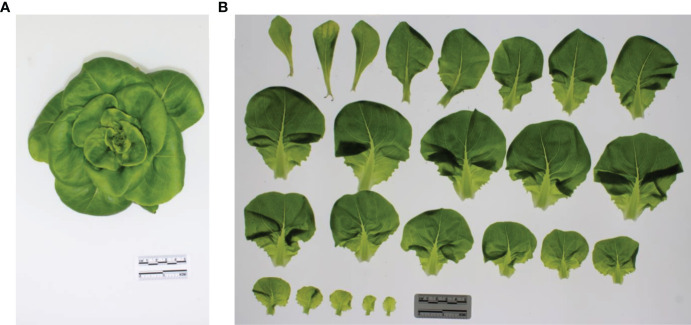
**(A)** Top view image of a lettuce plant grown under FR00 and harvested 14 DAT. **(B)** Picture of the same lettuce plant after the leaves longer than 1 cm were placed under a transparent glass plate for the determination of leaf area, length and width. The leaves are placed from left to right and from top to bottom in order of emergence.


Of all plants harvested, the number of leaves longer than 1 cm was counted and the fresh weight (FW) was determined for the whole lettuce shoot (leaves + stem) and the lettuce leaves separately. At 14 DAT, a top view image (**Figure 1A**) was taken of two plants per unit using a Canon EOS 2000D digital camera (Canon, Tokyo, Japan). The leaves were subsequently removed from the stem and placed on a LED backlit panel (RS Components Ltd., Corby, United Kingdom), covered with a transparent glass plate and also photographed (**Figure 1B**). The projected head area, as well as the area, length and width of each individual leaf, were determined by image analysis (ImageJ 1.53 software, National Institutes of Health, USA). The total leaf area was estimated as the sum of the individual leaves and the specific leaf area (SLA) is calculated here as the ratio of the total leaf area to the leaf dry weight. Then, the samples were dried at 70°C for 72 hours for dry weight (DW) determination. At 21 DAT, the harvesting procedure was performed as described above for one plant per unit. A second plant was harvested around eight hours after the start of the photoperiod and used for chemical analysis. The 16^th^, 17^th^ and 18^th^ true leaves of these plants were selected because they fully developed under the light treatments. These leaves were cut in half along the midrib, after which one half was considered as fresh leaf sample, while the other half was dried at 70°C for 72 hours for nitrate analysis. The three fresh leaf halves were pooled per sample, ground in liquid nitrogen with pestle and mortar and stored at -80°C until analysis. The remaining plants were harvested 28 DAT and the total leaf number, fresh and dry weight were determined. In order to take the possible effects of deviations in PAR flux into account, light use efficiency per mol incident photosynthetic photons (LUE_inc, PAR_) was calculated as

$$\text{LU}{\text{E}}_{\text{i}\text{n}\text{c},\text{P}\text{A}\text{R}}(\text{g mo}{\text{l}}^{-1})=\frac{\text{shoot }\text{d}\text{r}\text{y }\text{w}\text{e}\text{i}\text{g}\text{h}\text{t }(\text{g}\text{ p}\text{l}\text{a}\text{n}{\text{t}}^{-1})\text{ x}\text{ m}\text{e}\text{a}\text{n}\text{ p}\text{l}\text{a}\text{n}\text{t}\text{ d}\text{e}\text{n}\text{s}\text{i}\text{t}\text{y}\text{ o}\text{v}\text{e}\text{r}\text{ g}\text{r}\text{o}\text{w}\text{i}\text{n}\text{g}\text{ p}\text{e}\text{r}\text{i}\text{o}\text{d}\;(\text{p}\text{l}\text{a}\text{n}\text{t}\text{s}{\text{m}}^{-2})}{\text{c}\text{u}\text{m}\text{u}\text{l}\text{a}\text{t}\text{i}\text{v}\text{e}\text{ d}\text{a}\text{i}\text{l}\text{y}\text{ l}\text{i}\text{g}\text{h}\text{t}\text{ int}\text{e}\text{g}\text{r}\text{a}\text{l }[400-700\;\text{n}\text{m}]\text{ sin}\text{c}\text{e}\text{ t}\text{r}\text{a}\text{n}\text{s}\text{p}\text{l}\text{a}\text{n}\text{t}\text{i}\text{n}\text{g}\;(\text{m}\text{o}\text{l}{\text{m}}^{-2})}$$

### 2.3   Leaf optical properties

Leaf optical properties and vegetation indices were measured 20 days after the beginning of the light treatments on three leaves per plant and six plants per light treatment using a PolyPen RP 410 UVIS (PSI, Photon Systems Instruments, Drasov, Czech Republic). This device has a spectral response range from 380 to 790 nm. Leaf reflectance was calculated as $\text{R}=\text{I}/{\text{I}}_{0}$ , where I is the reflectance signal from the sample and I_0_ represents the light reflectance of a Spectralon calibration standard. Based on leaf reflectance, five vegetation indices were calculated as shown in **Supplementary Table 1.**

### 2.4 Phytochemical analyses

#### 2.4.1 Photosynthetic pigment content

Photosynthetic pigments were analysed according to Lichtenthaler and Buschmann (2001). Chlorophyll a (Chl_a_), chlorophyll b (Chl_b_) and carotenoids (C_x+c_) were extracted by incubating ± 90 mg leaf material (harvested 21 DAT) in 10 mL of acetone (80 vol%) for 24 hours at -20°C. After centrifugation for 10 minutes at 4000 rpm (Centrifuge 5804 R, Eppendorf, Hamburg, Germany), 200 µL of the supernatant was transferred to a 96-well plate and the absorbance was measured at 470 nm, 647 nm and 663 nm with a spectrophotometer (Infinite 200, Tecan Group Ltd., Männedorf, Switzerland) and adjusted for pathlength using the Lambert-Beer law. The pigment contents were calculated in µg per mL using the equations listed below and subsequently recalculated to µg per cm^2^ leaf area, using the specific leaf area on FW base. The total chlorophyll concentration was calculated as the sum of the chlorophyll a and b contents.


$$Chl_{a}=12.25\;\times A_{663}-2.79\times A_{647}$$

$$Chl_{b}=21.5\times A_{647}-5.1\;\times \;A_{663}$$

$$C_{x+c}=\left(1000\times A_{470}-1.82\times Chl_{a}-85.02\times Chl_{b}\right)/198$$

#### 2.4.2 Total phenolics content

Total phenolics were determined spectrophotometrically as described by Liu et al. (2007). ± 200 mg leaf material was extracted with 80 vol% MeOH and sonicated for 30 minutes at room temperature. Next, the samples were centrifuged for 5 minutes at 11000 rpm and 20 µL of the supernatant was transferred to a 96-well plate. 100 µL Folin-Ciocalteu reagent (1:10 dilution) and 80 µL Na_2_CO_3_ (75 g/L) were added, followed by incubation in the dark for 2 hours at room temperature. The absorbance of the reaction product was measured at 765 nm (Infinite 200, Tecan Group Ltd., Männedorf, Switzerland) and the concentration of total phenolics was expressed as µg gallic acid per g DW (µg GAE/g DW) using the average dry matter percentage per treatment obtained at 21 DAT.

#### 2.4.3 Soluble carbohydrates content

Carbohydrates were extracted according to Christiaens et al. (2015) from ± 250 mg leaf material in 80% ethanol at 45°C for 3 hours, followed by centrifugation at 5000 rpm for 5 min (Centrifuge 5804 R, Eppendorf, Hamburg, Germany). Glucose, fructose and sucrose were separated with a CarboPac PA-20 analytical column and companion guard column (Thermo Fisher Scientific, Sunnyvale, CA, USA) and an eluent of 50 mM NaOH at 35°C. Subsequently, these soluble carbohydrates were quantified by high-pressure liquid chromatography with pulsed amperometric detection (ACQUITY UPLC H-Class, Waters, Milford, MA, USA) and recalculated to mg sugar/g DW, using the average dry weight percentage per treatment obtained at 21 DAT.

#### 2.4.4 Nitrate content

The nitrate concentration was determined colorimetrically as described by Cataldo et al. (1975). Nitrate was extracted by suspending ±100 mg of oven-dried samples in demineralized water for 3 hours at 45°C. 2 µL of the extract was transferred to a 96-well plate, after which 8 µL of a 5% (w/v) solution of salicylic acid in concentrated H_2_SO_4_ was added. After 20 minutes, 190 µL of 4 M NaOH was added and the absorbance was measured at a wavelength of 410 nm (Infinite 200, Tecan Group Ltd., Männedorf, Switzerland). The nitrate concentration was calculated according to a calibration curve using NaNO_3_ and expressed as mg nitrate per g FW, using the average dry matter percentage per treatment obtained at 21 DAT in order to compare the results to norms set by the European Commission (2011).

### 2.5 Statistical analysis

Spectrophotometric analyses were performed in triplicate and averaged for each sample. All statistical analysis was conducted in RStudio (R version 4.2.2, R Core Team, Vienna, Austria), extended with the *agricolae* (de Mendiburu, 2021), *car* (Fox and Weisberg, 2019), *DescTools* (Signorell, 2022), *egg* (Auguie, 2019), *ggbiplot* (Vu, 2011), *ggbreak* (Xu et al., 2021) and *ggplot2* packages (Wickham, 2016). Differences at the 5% level were considered statistically significant. Normality and homoscedasticity of the data were assessed by using a Shapiro-Wilk and Levene test, respectively. If both assumptions were met, one-way ANOVA was performed, followed by Tukey’s HSD for *post-hoc* comparisons. LUE_inc,PAR_ was analysed using linear regression with the far-red fraction as the independent variable. For leaf area and dimensions, a Kruskal-Wallis test was performed to indicate statistical differences. Principal component analysis (PCA) was conducted to determine whether there was a clear clustering of light quality treatments and to identify which vegetation indices were most discriminative. Pearson’s correlation coefficient was used to examine the correlation between total sugars and nitrate content.

## 3 Results

### 3.1 Plant morphology

**Figure 2:**
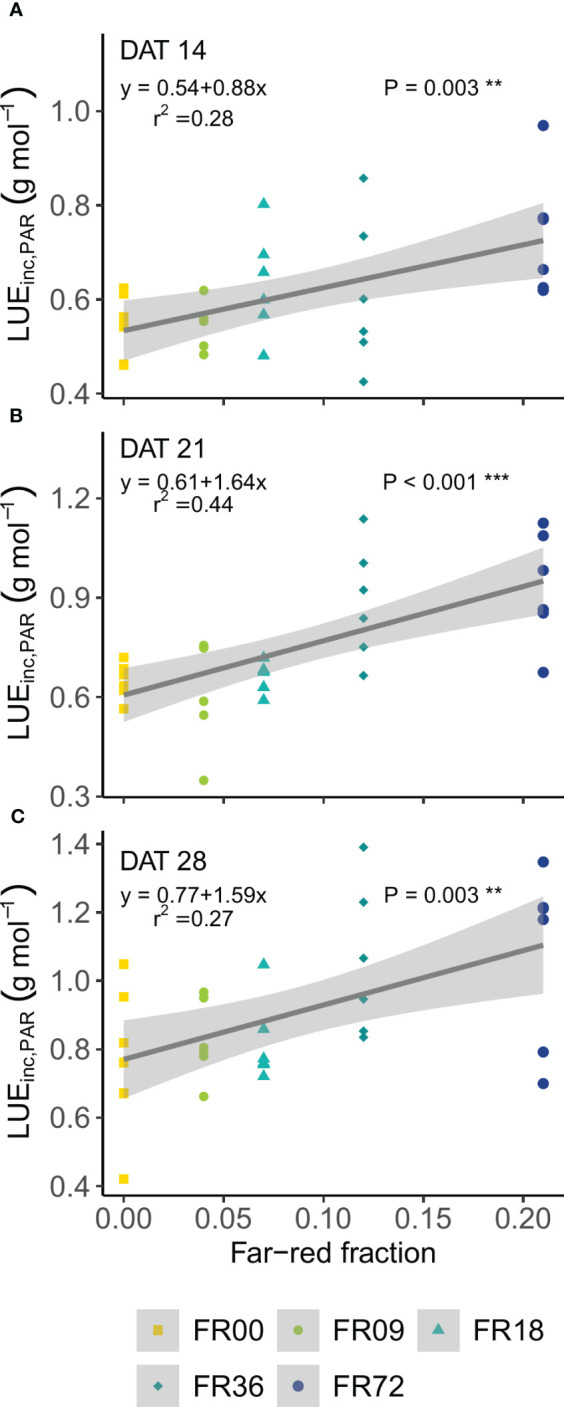
Effect of far-red fraction on light use efficiency, calculated on PAR, at **(A)** 14 DAT, **(B)** 21 DAT and **(C)** 28 DAT. ** indicates significance at *P* < 0.01 and *** indicates significance at *P* < 0.001.

**Figure 3:**
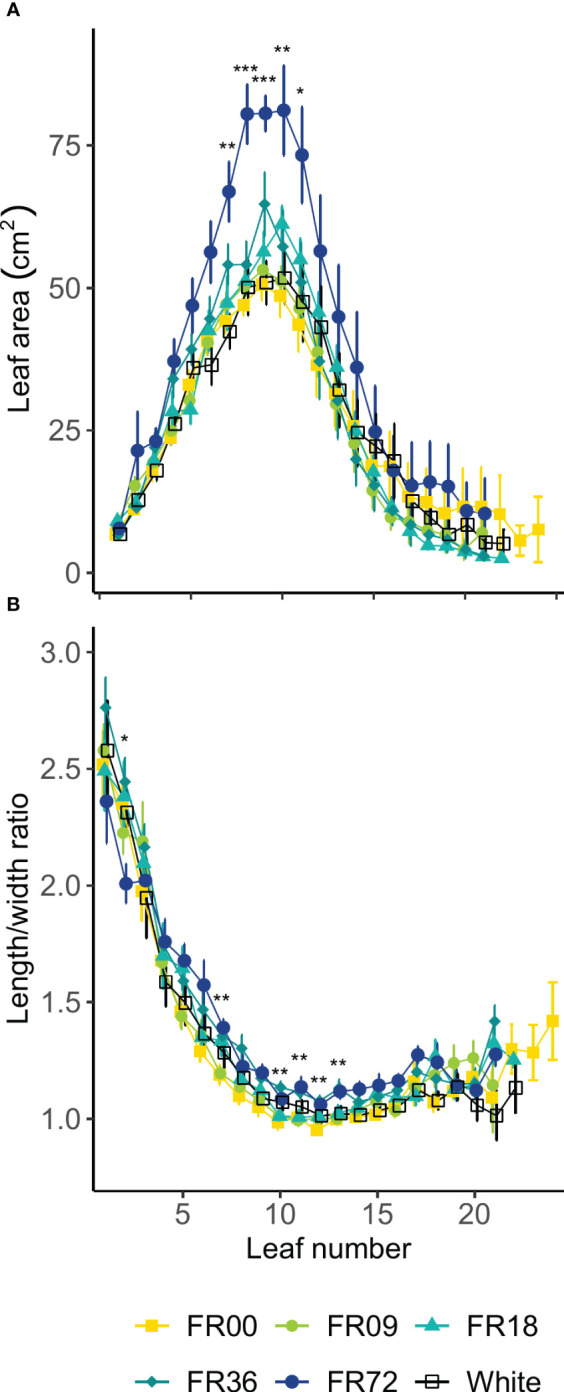
Effect of far-red addition on **(A)** lettuce leaf area and **(B)** length-to-width ratio in function of leaf development, lowest numbers corresponding to the oldest true leaves, measured 14 DAT. Values shown are means ± SE, n ≥ 3. Different symbols mark significant differences between light quality treatments based on a Kruskal-Wallis test, * indicates significance at *P* < 0.05, ** indicates significance at *P* < 0.01 and *** indicates significance at *P* < 0.001.

**Table 2** shows the total shoot fresh and dry weight, leaf number, projected head area and specific leaf area of plants grown under the different light treatments described in **Table 1**. The average shoot FW increased from 27.8 to 131.7 g between 14 and 28 DAT, while the shoot DW increased from 1.82 to 7.94 g. However, there were deviations in PAR photon flux between the treatments and thus the effects on biomass were not solely caused by far-red addition. Therefore, we focus on LUE_inc,PAR_ because this metric corrects for the deviations in PAR. The effects of far-red fraction on LUE_inc,PAR_ were analyzed by linear regression and the results are shown per harvest date in **Figure 2**. LUE_inc,PAR_ ranged from 0.35 to 1.39 g DW mol^-1^, depending on harvest date and far-red treatment, and increased over time for all treatments, as can be seen in the regression intercepts. The regression coefficients were highly significant (*P *< 0.01 or *P *< 0.001) for all harvest dates, showing a positive effect of far-red fraction on light use efficiency. Compared to the light treatment with no addition of far-red photons (FR00), a far-red fraction of 0.21 increased the mean LUE_inc,PAR_ by 37% at 14 DAT, 43% at 21 DAT and 39% at 28 DAT. The average leaf number increased from 18.7 to 36.3 leaves per plant between 14 and 28 DAT. The projected head area increased significantly at a higher far-red photon flux, both for plants harvested 14 DAT and 21 DAT. When comparing the treatments with the lowest (FR00) and highest (FR72) far-red doses, the projected head area increased by 71% and 66% at 14 and 21 DAT, respectively. The specific leaf area showed a significant increase of about 50 cm^2^ g^-1^ for plants grown under FR36 and FR72 in comparison with the other treatments. Leaf area is given in function of leaf number in **Figure 3A**, with low leaf numbers corresponding to the first developed true leaves. The effect of far-red addition on leaf expansion was most apparent for leaves 5-13, with higher far-red fractions increasing the leaf area. The area increase was mostly due to a stronger expansion in the direction of leaf length, while the effect of far-red addition on leaf width was more limited (**Supplementary Figures 2A, B**). This results in a higher length-to-width ratio for plants grown under FR36 and FR72, as shown in **Figure 3B**. For the first ten leaves, the length-to-width ratio decreases in function of leaf ontogeny, with the tenth leaf having the lowest length-to-width ratio. However, this decrease in length-to-width ratio seems to lag behind in treatments with higher far-red photon fluxes, as similar ratios are reached about two leaves later compared to the plants grown under a low far-red photon flux. Total leaf area and total leaf DW were linearly correlated for the different light qualities, with high coefficients of determination (r^2^ > 0.95) for all treatments (**Supplementary Figure 3**). The regression curves of the FR00, FR09, FR18 and White treatments almost coincide, indicating a similar specific leaf area (SLA) among these treatments, whereas FR36 and FR72 showed a higher specific leaf area, confirming the findings from **Table 2**.

**Table 2:** Effect of far-red addition on shoot FW, shoot DW, lettuce leaf number, projected head area and specific leaf area measured at different timepoints.

DAT	Light treatment code	Red/ far-red ratio	PSS	Far-red fraction	Shoot FW (g)		Shoot DW (g)		Total leaf number (n plant^-1^)		Projected head area (cm^2^)		SLA (cm^2^ g^-1^ DW)	
14	FR00	∞	0.886	0.00	26.1 ± 1.8	a	1.70 ± 0.09	ab	19.4 ± 0.9	a	185.0 ± 18.7	b	280.6 ± 11.9	b
	FR09	29.29	0.874	0.04	27.3 ± 0.9	a	1.77 ± 0.03	ab	18.6 ± 0.4	a	184.5 ± 16.6	b	280.0 ± 6.3	b
	FR18	15.61	0.863	0.07	30.3 ± 2.1	a	2.01 ± 0.13	ab	19.2 ± 0.7	a	222.9 ± 20.9	b	273.4 ± 9.3	b
	FR36	8.12	0.844	0.12	24.6 ± 3.6	a	1.53 ± 0.17	b	17.3 ± 1.1	a	252.8 ± 77.6	ab	339.4 ± 12.7	a
	FR72	4.27	0.809	0.21	32.9 ± 2.5	a	2.18 ± 0.16	a	18.6 ± 0.7	a	316.9 ± 39.0	a	335.0 ± 10.2	a
	White	4.62	0.828	0.18	25.4 ± 2.5	a	1.74 ± 0.15	ab	18.8 ± 0.8	a	203.9 ± 30.8	b	282.8 ± 8.1	b
	*P*-value				0.13		0.02	*	0.51		< 0.001	***	< 0.001	***
21	FR00	∞	0.886	0.00	58.5 ± 4.6	a	3.67 ± 0.14	b	29.0 ± 0.9	a	279.1 ± 47.1	b		
	FR09	29.29	0.874	0.04	59.7 ± 6.8	a	3.61 ± 0.34	b	28.3 ± 1.0	a	278.1 ± 55.0	b		
	FR18	15.61	0.863	0.07	61.1 ± 3.8	a	3.82 ± 0.18	ab	27.8 ± 0.9	a	304.4 ± 49.9	b		
	FR36	8.12	0.844	0.12	66.2 ± 5.8	a	4.01 ± 0.34	ab	27.2 ± 1.2	a	467.2 ± 52.0	a		
	FR72	4.27	0.809	0.21	79.9 ± 7.8	a	4.94 ± 0.35	a	28.5 ± 1.1	a	463.3 ± 77.5	a		
	White	4.62	0.828	0.18	60.5 ± 2.6	a	3.77 ± 0.19	b	28.3 ± 1.2	a	322.7 ± 50.6	b		
	*P*-value				0.09		0.02	*	0.88		< 0.001	***		
28	FR00	∞	0.886	0.00	127.7 ± 17.1	a	7.26 ± 0.70	a	38.2 ± 2.4	a				
	FR09	29.29	0.874	0.04	137.2 ± 7.1	a	8.01 ± 0.35	a	38.2 ± 1.4	a				
	FR18	15.61	0.863	0.07	131.7 ± 8.1	a	7.79 ± 0.31	a	35.2 ± 0.6	a				
	FR36	8.12	0.844	0.12	133.0 ± 9.6	a	7.84 ± 0.44	a	34.3 ± 1.2	a				
	FR72	4.27	0.809	0.21	143.3 ± 14.7	a	9.41 ± 0.75	a	34.7 ± 2.0	a				
	White	4.62	0.828	0.18	117.1 ± 17.6	a	7.35 ± 0.65	a	37.0 ± 2.2	a				
	*P*-value				0.80		0.12		0.44					

Data shown are means ± SE, n = 6. Different symbols mark significant differences between light quality treatments based on a One-Way ANOVA, * indicates significance at *P* < 0.05, ** indicates significance at *P* < 0.01 and *** indicates significance at *P* < 0.001. Different letters indicate statistical differences between light quality treatments based on Tukey’s HSD (*P* = 0.05).

### 3.2 Leaf optical properties

**Figure 4:**
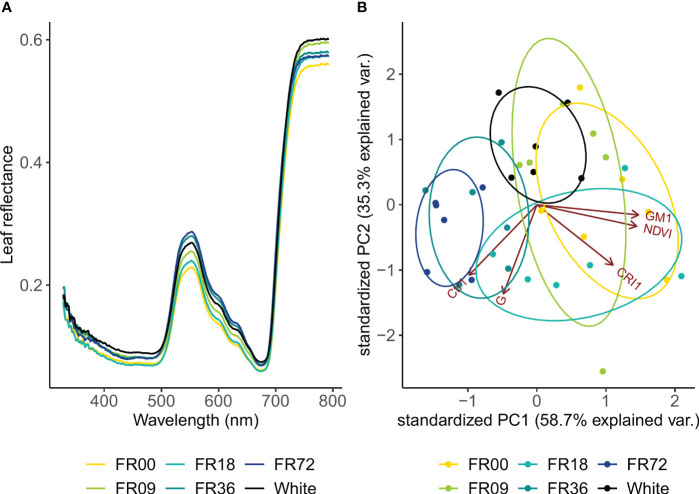
**(A)** Leaf reflectance pattern of lettuce cv. Alyssa under different far-red photon fluxes. Values shown are mean values per light treatment (n = 6). **(B)** PCA biplot of individual samples to PC 1 and PC 2. Ellipses denote 95% confidence interval of light treatments.

Leaf reflectance showed the largest differences between light treatments in the green wavelengths and the NIR region (**Figure 4A**). In general, reflectance in the green wavebands was higher in plants grown under higher far-red intensities, except for the FR18 treatment, which showed reflectance values between those of the FR00 and FR09 treatments. At the peak reflectance of 555 nm, the reflectance was 25% higher for FR72 compared to FR00. In the NIR region, leaf reflectance did not show a clear trend according to far-red addition. Based on these reflectance patterns, five vegetation indices were calculated (**Supplementary Table 2**) and subsequently analysed by PCA. The first two components of the PCA analysis explained 94.0% of the variance in the vegetation index dataset (PC 1: 58.7%, PC 2: 35.3%; **Figure 4B**). PC1 had a strong positive correlation with GM1, NDVI and CRI1 but a strong negative correlation with Ctr1. PC2 had the strongest negative correlation with G, Ctr1 and CRI1. Light treatments show clear clusters, as denoted by the 95% confidence interval ellipses, which tend to shift towards the left side of the PC1 axis with increasing far-red doses.

### 3.3 Phytochemical analyses

**Figure 5:**
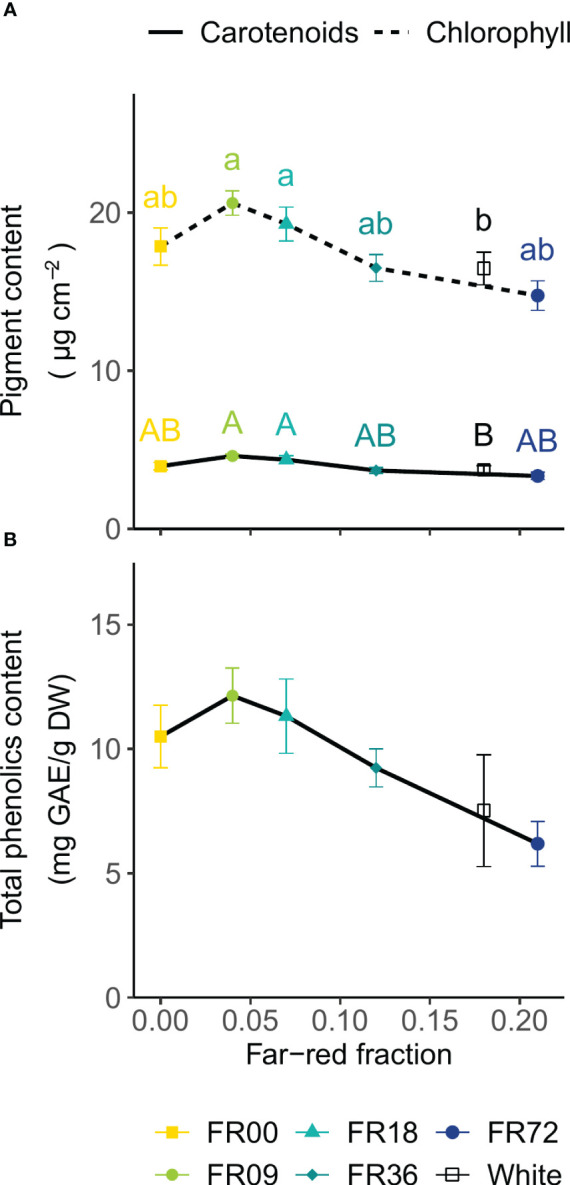
Effect of far-red fraction on **(A)** leaf carotenoid and total chlorophyll content and **(B)** leaf total phenolics content of lettuce cv. Alyssa, harvested 21 DAT. Values shown are means ± SE, n = 6. Different letters indicate statistical differences between light quality treatments based on Tukey’s HSD (*P* = 0.05). If no letters are displayed, no statistical differences were found based on ANOVA.

For all light quality treatments, leaf chlorophyll and carotenoid concentrations are presented in **Figure 5A** as a function of their far-red fraction. The chlorophyll content showed a decreasing trend with increasing far-red fraction, with FR09 (20.6 ± 0.8 µg per cm^2^, far-red fraction 0.04) containing the highest concentration and FR72 (far-red fraction 0.21) the lowest concentration (14.8 ± 0.9 µg per cm^2^). A very similar trend is visible for the carotenoid content, with a minimum of 3.3 ± 0.1 µg per cm^2^ for FR72 and a maximum of 4.6 ± 0.1 µg per cm^2^ for FR09. For the total phenolics content of the leaves, a decreasing trend is discernable with higher far-red fractions (**Figure 5B**). The total phenolics content ranged from 6.2 ± 0.9 mg GAE/g DW (FR72) to 12.1 ± 1.1 mg GAE/g DW (FR09). Although One-Way ANOVA showed significant differences (*P* = 0.04), neither Tukey’s HSD nor Dunnett’s test was able to discriminate between treatments. The white LED treatment had a similar pigment and total phenolics content to FR72, which has a similar far-red fraction.

**Figure 6:**
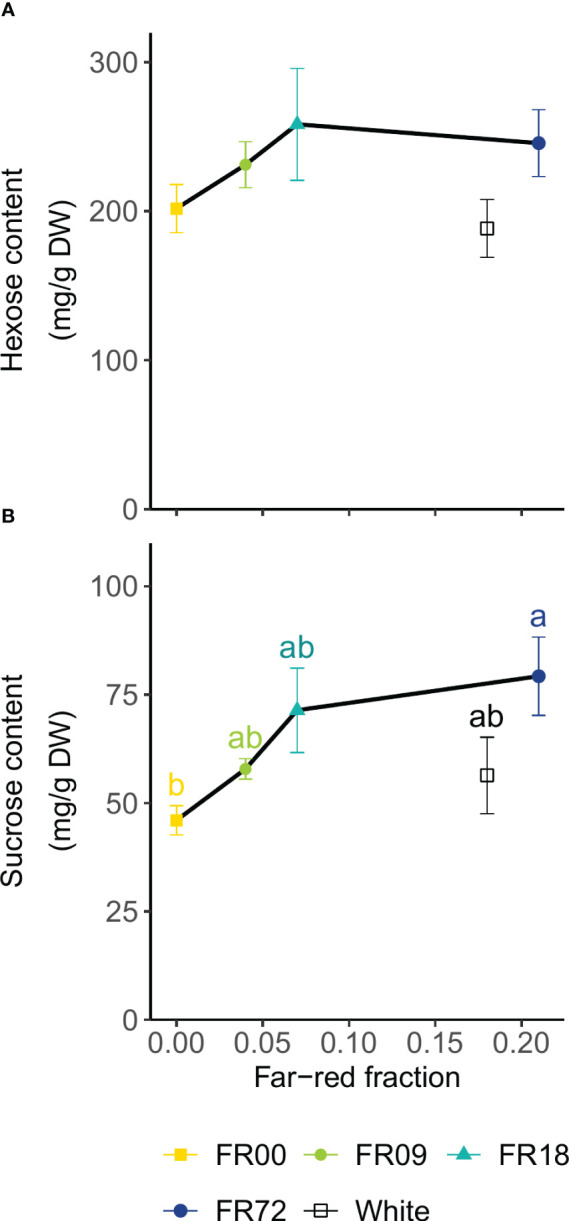
Effect of far-red fraction on **(A)** leaf hexose content (glucose + fructose) and **(B)** leaf sucrose content of lettuce cv. Alyssa, harvested 21 DAT. Values shown are means ± SE, n = 6. Different letters indicate statistical differences between light quality treatments based on Tukey’s HSD (*P* = 0.05). If no letters are displayed, no statistical differences were found based on ANOVA.

**Figure 6** displays the results of the soluble carbohydrate analysis as a function of far-red fraction. The FR36 treatment was omitted because of the deviation in PAR flux compared to the other treatments (**Table 1**). The hexose content (calculated as the sum of glucose and fructose contents) did not significantly differ among treatments (**Figure 6A**). The sucrose contents increased with increasing far-red fractions. Statistical differences were found between FR72 (79.3 ± 9.0 mg sucrose/g DW, far-red fraction 0.21) and FR00 (46.0 ± 3.3 mg sucrose/g DW, far-red fraction 0.00) (**Figure 6B**). In both cases, the plants grown under white LEDs showed a lower sugar concentration than the far-red treatment with a similar far-red fraction (FR72).

**Figure 7:**
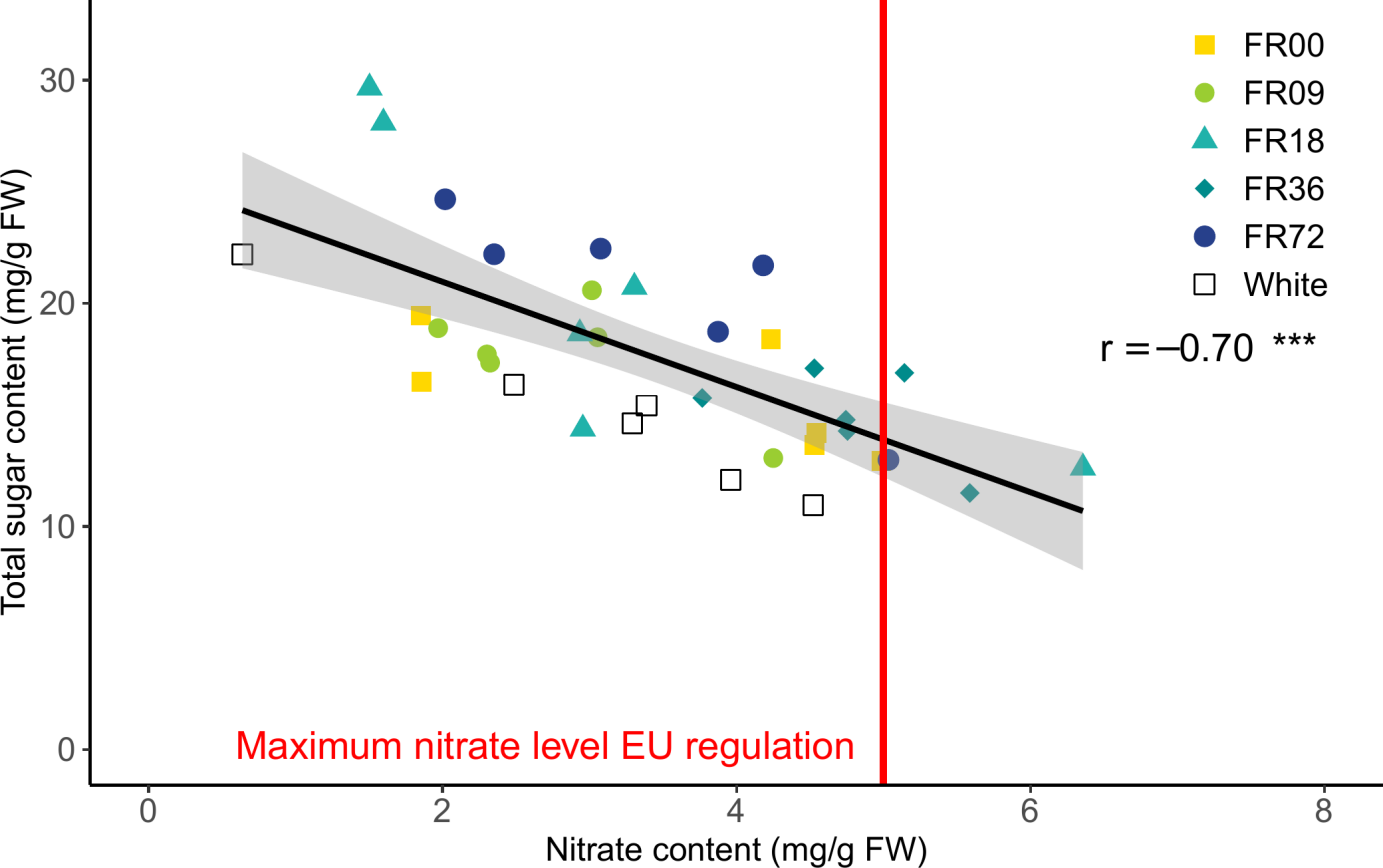
Correlation between leaf nitrate and total sugar content for lettuce cv. Alyssa, harvested 21 DAT. The gray area indicates the 95% confidence interval, while the vertical red line represents the maximum nitrate level during winter allowed by the European Commission (2011). The overall Pearson correlation r is shown, along with its significance level. *** indicates significance at *P* < 0.001.

The effect of the light quality treatments on nitrate content are presented in **Supplementary Figure 4.** Although a high variability in total sugar content (glucose + fructose + sucrose) and nitrate content is present for most light quality treatments, a strong negative correlation was found between these metabolites (*r* = -0.70, **Figure 7**). Moreover, it is noteworthy that plants grown under FR36, which thus received a lower PAR flux, have a higher nitrate content and relatively low sugar content compared to the other treatments. Overall, four datapoints exceeded the maximum allowed concentration of nitrate (5,000 mg nitrate per kg FW or 5 mg nitrate per g FW), as set by the European Commission for lettuce grown during the winter in protected cultivation systems (European Commission, 2011).

## 4 Discussion

Far-red photon flux is widely known to induce stem elongation and leaf expansion to maximize light interception (Smith and Whitelam, 1997; Franklin, 2008; Demotes-Mainard et al., 2016; Wong et al., 2020). High far-red fractions, generated by addition of far-red photons in horticultural light recipes, may therefore increase leaf area and light interception and eventually biomass accumulation. Increased above-ground plant growth has indeed been observed by other authors. For instance, Jin et al. (2021) found higher fresh and dry weight for leaf lettuce grown at three planting densities when 50 µmol m^-2^ s^-1^ of far-red was added to the spectrum, with a higher effect at the lowest density. In the present research, the PAR flux deviated from the 200 µmol m^-2^ s^-1^ setpoint, therefore the LUE_inc,PAR_ is the most appropriate metric to assess the influence of the applied far-red fluxes on biomass accumulation.The far-red fraction significantly increased LUE_inc,PAR_ at all harvest dates, confirming earlier findings. In addition, LUE_inc,PAR_ increased over time, which can be explained by a better interception of light energy due to the increase in leaf area. Similar LUE_inc,PAR_ values to the ones obtained in our trial and a similar increase in LUE_inc,PAR_ over time are reported by Jin et al. (2022). So far, there are only a few studies available on interactive effects of PAR and far-red flux on lettuce growth. Legendre and Iersel (2021) reported no interactive effects between PAR flux and far-red photon flux, while Kusuma and Bugbee, (2023) found a positive effect of far-red on leaf area and dry mass accumulation at high PAR fluxes, but a negative effect on these growth parameters at lower PAR fluxes. Interactive effects between PAR flux and far-red fractions have previously been described for other crops (e.g. lentil,Yuan et al., 2017; and soybean, Hitz et al., 2019). Furthermore, these responses may be cultivar- and even organ-dependent. Therefore, more research is required to disentangle the effects of PAR and far-red flux on lettuce and their possible interaction.

The total leaf number was not influenced by the far-red fraction in our study, although it has previously been reported that high fractions may reduce the number of leaves per plant for leaf and romaine lettuce (Lee et al., 2015; Kong and Nemali, 2021). We did, however, observe a significant increase in leaf area and projected head area for plants grown under high far-red fractions, which confirms that light capture was enhanced by far-red addition at the early growth stages. The main parameter driving the increase in leaf area was leaf length, which was significantly higher for plants grown under high far-red fractions. This increase in leaf size is likely caused by an increase in cell expansion, as the PAR flux was sufficiently high to provide an adequate carbon supply for plant growth (Park, 2017). According to Bensink (1971), the onset of lettuce head formation is characterized by a drop in the leaf length-to-width ratio below 1. In our trial, the minimal ratios obtained were higher than 1, with significant differences between far-red treatments. Low length-to-width ratios were obtained about two leaves earlier for low far-red fractions, which may indicate that far-red addition postpones the start of head formation. Specific leaf area was reduced at high far-red fractions, implying that the leaves were thinner. This is also a known effect of far-red addition (Franklin, 2008).

In addition to phytochrome-mediated responses, far-red also plays a critical role in photosynthesis. Although PAR is traditionally defined as the photon flux from 400 to 700 nm (McCree, 1972), recent findings prove that far-red photons in the 700-750 nm range are equally efficient to drive photosynthesis, because they act in synergy with the traditionally defined PAR photons by preferentially stimulating photosystem I and thus restoring the excitation balance between photosystem I and II (Zhen et al., 2021). Although no photosynthesis measurements were performed in the experiment presented in the present study, it is beyond dispute that the effects of the increased far-red fraction on dry weight accumulation and related parameters can be partly attributed to the restored excitation balance between photosystem I and II.

It is striking that the plants grown under white LEDs perform very differently compared to the far-red treatment with a similar far-red fraction (FR72). However, this can be explained by the relatively low amount of red radiation present in these LED fixtures. In our trial, the white LED treatment consisted of about 200 µmol m^-2^ s^-1^ PAR flux (20.8% blue, 39.7% green, 39.5% red) and about 13 µmol m^-2^ s^-1^ far-red. On the other hand, the far-red treatments consist of about 200 µmol m^-2^ s^-1^ PAR flux (10.0% blue, 90.0% red), supplemented with different far-red doses. In other words, the white LEDs provide a large amount of green radiation at the expense of red radiation. This results in a high far-red fraction, but at the same time, a large quantity of photosynthetically very efficient red photons is substituted by green photons, which are less efficient for photosynthesis (McCree, 1972). This may partly explain the differences in plant morphology obtained for plants grown under white LEDs.

Leaf reflectance patterns mainly differed between treatments in the green wavelengths (500-600 nm). High reflectance in this region is associated with low chlorophyll contents (Maes and Steppe, 2019), indicating that far-red addition led to lower chlorophyll concentrations in our trial. This is confirmed by the PCA analysis of the vegetation indices, as the far-red doses show a negative correlation with chlorophyll-related indices (NDVI and GM1). This negative correlation between chlorophyll concentration and far-red photon flux is confirmed in the destructive pigment analysis and corresponds with observations found in literature, where most authors report a lower chlorophyll content under far-red supplementation (Meng and Runkle, 2019; Wong et al., 2020; Kong and Nemali, 2021). Zou et al. (2019) also observed lower chlorophyll contents under far-red addition and suggests that the increase in specific leaf area under high far-red fractions lowers the chlorophyll content per unit of leaf area. This “dilution effect” is thus supported by our results for head lettuce, by both chemical analysis and the GM1 index. A similar explanation may be given for the carotenoid content, as low far-red fractions corresponded with low CRI1 values and destructive analysis also indicated lower carotenoid concentrations for the highest far-red fraction. A decrease in carotenoid concentration under high far-red fractions was previously described by other authors, including Li and Kubota (2009) and Mickens et al. (2018). The main function of plant pigments, such as chlorophyll and carotenoids, is to absorb light energy and transfer it by resonance to the reaction centre pigments, which initiates the photosynthetic process (Richardson et al., 2002). The decrease in specific pigment content indicates that, however leaf area is increased under high far-red fractions, the plants may not reach their full potential in terms of photon capture, and this may thus be a limiting factor for further increasing photosynthetic performance. Furthermore, the PCA analysis revealed that light recipes rich in far-red led to an increase in the Ctr1 index, which is related to plant stress (Carter, 1994). This may also partly explain the limited effect of far-red on fresh biomass accumulation in the present trial.

Destructive analyses of total polyphenols showed an decreasing trend with increasing far-red fractions. Polyphenols are produced in the shikimate pathway (Tsao, 2010) and contribute to the plant’s defense against insects, microbial pathogens and fungi (Ballaré, 2014). Therefore, our results indicate that plants grown under a higher far-red photon flux may be more susceptible to pests and pathogens. This effect of far-red light has previously been observed, for instance for *Manduca sexta* caterpillars feeding on *Nicotiana longiflora* (Izaguirre et al., 2006) and for *Botrytis cinerea* growing on *Arabidopsis thaliana* (Cerrudo et al., 2012). From a nutritional point of view, the decrease in polyphenols under high far-red fractions may also be undesired, as these compounds are considered to have antioxidant properties (Vuolo et al., 2018).

Soluble carbohydrates are an important factor in determining sensorial quality, improving sweetness and crispness of lettuce (Lin et al., 2013). Moreover, high levels of carbohydrates are often associated with an improved shelf life of the produce (Woltering and Witkowska, 2016). In this study, we found an increasing trend for hexose and sucrose contents with increasing far-red fractions. Higher soluble sugar levels were also reported by Zou et al, 2019; Zou et al, 2021), but it is not clear which pathways are involved and the effect of far-red on assimilation products may be species-dependent (Tan et al., 2022). Zou et al. (2019) attribute the enhanced soluble sugar content under far-red illumination to an increased activity of sucrose phosphate synthase. This catalyzes the synthesis of sucrose from its glucose and fructose precursors (Huber and Huber, 1996). This enhancement of sucrose phosphate synthase through phytochrome activity has also been demonstrated in other crops, such as radish (Keiller and Smith, 1989) and mustard (Yanovsky et al., 1995), but it does not explain the increase in hexose content observed in the present study. However, as has been mentioned above, far-red photons improve photosynthetic efficiency by restoring the excitation balance between photosystem I and II. Therefore, increased photosynthesis may explain the enhanced concentration of photosynthetic products found in lettuce grown high far-red fractions. The lower sugar contents for plants grown under white LEDs in comparison to the FR72 treatment can be attributed to the substitution of photosynthetically efficient red radiation by less efficient green radiation, in a similar way to the differences in morphologic parameters.

Lettuce is known to accumulate nitrate in the vacuole as an osmoticum to maintain turgor pressure in cells when concentrations of other organic compounds, such as carbohydrates and organic acids, are limited (Behr and Wiebe, 1992). In this study, we indeed observed a strong negative correlation between nitrate and soluble carbohydrate levels, but no significant differences in nitrate content were found between far-red treatments with similar PAR flux. This is in agreement with findings by Zou et al. (2021). On the other hand, Li et al. (2021) observed a significant increase in nitrate concentration of red oakleaf and red butterhead lettuce when 10 µmol m^-2^ s^-1^ of far-red photons were added to ca. 245 µmol m^-2^ s^-1^ white LED light. The FR36 treatment, which received lower PAR flux in our trial, showed higher nitrate accumulation in comparison to the other treatments. This effect of light intensity has also been demonstrated by other researchers (e.g. Viršilė et al., 2020). However, excessive nitrate intake poses health risks. In the gastrointestinal tract, nitrate is converted by bacterial enzymes to nitrite, which can react with haemoglobin to form methaemoglobin, impairing oxygen transport in the blood. In addition, nitrite may react with amines or amides to form carcinogenic N-nitroso compounds (Santamaria, 2006; Hmelak Gorenjak and Cencič, 2013). Therefore, the European Union defined maximum levels of nitrate in lettuce, which vary depending on the harvesting period. For lettuce grown under cover, the limits are set at 4,000 or 5,000 mg nitrate kg^-1^ FW from 1 April to 30 September or from 1 October to 31 March, respectively (European Commission, 2011). In the present study, four plants (11% of total number of analyzed plants) exceeded the least restrictive limit and may therefore be retained from the European market.

## 5 Conclusion

The optimization of light recipes is key to improve crop production and quality in vertical farming facilities and to make these systems economically viable. In our experiment, we supplemented 200 µmol m^-2^ s^-1^ of PAR flux with five increasing intensities of far-red light. Consistent with previous studies, our results show that far-red supplementation up to 21% enhances photon capture and thus light use efficiency. Moreover, far-red supplementation tended to increase sugar content, which is a measure of organoleptic and postharvest quality. However, a high far-red photon flux resulted in a lower pigment content per leaf area and may alter metrics traditionally associated with plant stress, as indicated by vegetation indices. Furthermore, head formation may be delayed in butterhead lettuce. In conclusion, adding far-red light during the first phase of leaf initiation and development of butterhead lettuce may be beneficial for lettuce cultivation by improving light use efficiency. Once head formation starts the potential negative effects on leaf pigmentation and potential impact on nutritional and postharvest quality need to be taken into account.

## Data availability statement

The raw data supporting the conclusions of this article will be made available by the authors, without undue reservation.

## Author contributions

EV: Conceptualization, methodology, formal analysis, writing – original draft, visualization. KS: Project administration, funding acquisition, writing – review & editing. M-CV: Conceptualization, resources, supervision, writing – review & editing. All authors reviewed and approved the final manuscript.
