# Supplementary material for: Leaf morphology, optical characteristics and phytochemical traits of butterhead lettuce affected by increasing the far-red photon flux
Source: Front Plant Sci. 2023 Aug 2;14:1129335. doi: 10.3389/fpls.2023.1129335 (PMC10433762; doi:10.3389/fpls.2023.1129335)
Supplement: Supplementary file 1 [file DataSheet_1.pdf]

Supplementary Material

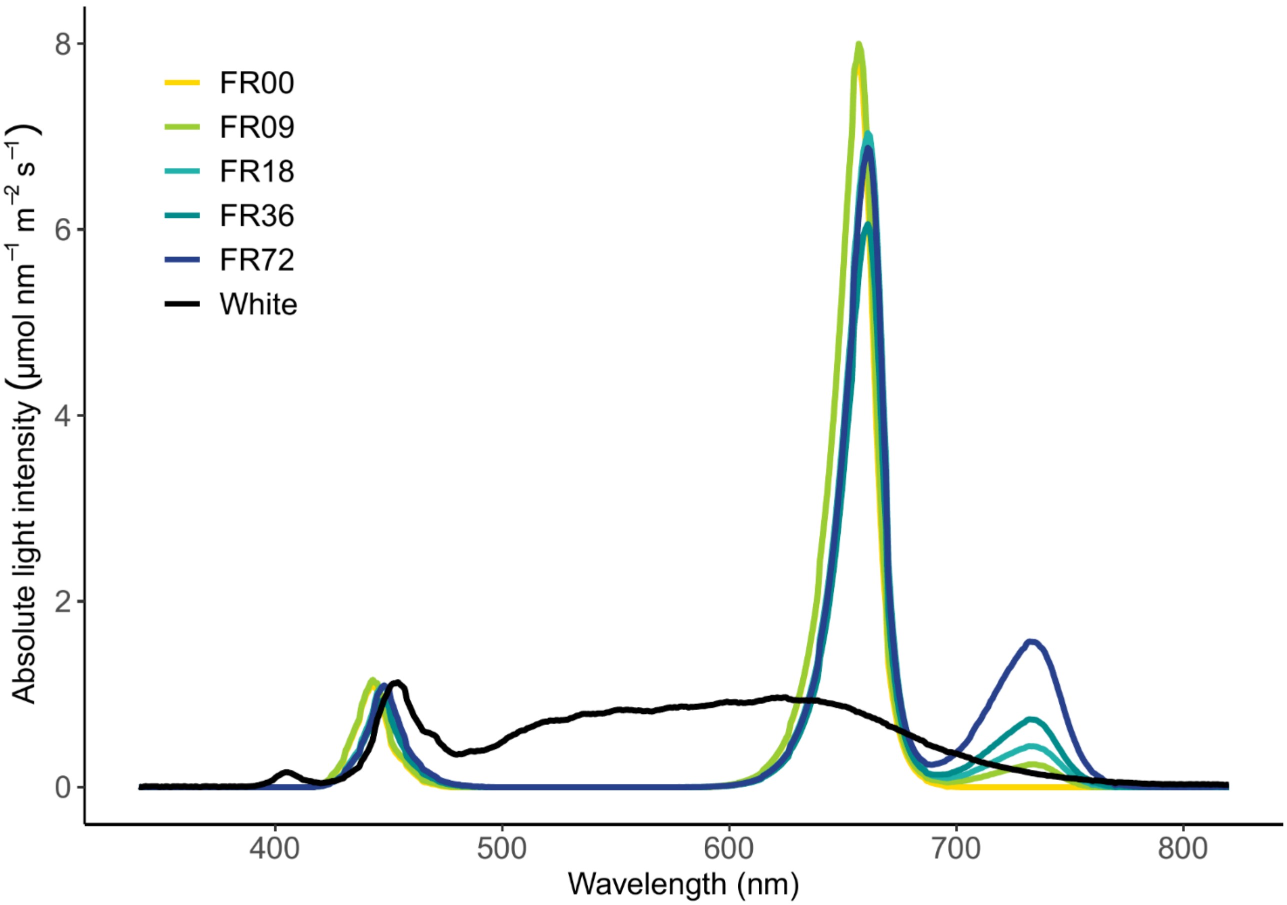

Supplementary Figure 1 Spectral distribution of the LED light used in this study, as measured by a SS-110 spectroradiometer (Apogee Instruments, Logan, UT, USA). Data shown are means of 10 measurements per treatment.

Supplementary Table 1 Calculation of selected vegetation indices based on leaf reflectance data, with R_λ representing the reflectance measured at λ nm.

Vegetation index	Abbreviation	Plant feature	Formula	Reference
Normalized Difference Vegetation Index	NDVI	General plant vigor	$\frac{R_{800} - R_{670}}{R_{800} + R_{670}}$	Rouse et al. (1974)
Greenness Index	G	Color	$\frac{R_{554}}{R_{677}}$	Zarco-Tejada et al. (2001)
Carter Index 1	Ctr1	Plant stress	$\frac{R_{695}}{R_{420}}$	Carter (1994)
Gitelson and Merzlyak Index 1	GM1	Chlorophyll content	$\frac{R_{750}}{R_{550}}$	Gitelson and Merzlyak (1997)
Carotenoid Reflectance Index 1	CRI1	Carotenoid/chlorophyll ratio	$\frac{1}{R_{510}} - \frac{1}{R_{550}}$	Gitelson et al. (2002)

References

Carter, G. A. (1994). Ratios of leaf reflectances in narrow wavebands as indicators of plant stress. *International Journal of Remote Sensing*, *15*(3), 517–520. https://doi.org/10.1080/01431169408954109

Gitelson, A. A., & Merzlyak, M. N. (1997). Remote estimation of chlorophyll content in higher plant leaves. *International Journal of Remote Sensing*, *18*(12), 2691–2697. https://doi.org/10.1080/014311697217558

Gitelson, A. A., Zur, Y., Chivkunova, O. B., & Merzlyak, M. N. (2002). Assessing carotenoid content in plant leaves with reflectance spectroscopy. *Photochemistry and Photobiology*, *75*(3), 272. https://doi.org/10.1562/0031-8655(2002)075<0272:accipl>2.0.co;2

Rouse, J., Haas, R. H., Scheel, J., & Deering, D. (1974). Monitoring vegetation systems in the Great Plains with ERTS. *3rd Earth Resource Technology Satellite (ERTS) Symposium, Vol. 1*, 48– 62.

Zarco-Tejada, P. J., Miller, J. R., Noland, T. L., Mohammed, G. H., & Sampson, P. H. (2001). Scaling-up and model inversion methods with narrowband optical indices for chlorophyll content estimation in closed forest canopies with hyperspectral data. *IEEE Transactions on Geoscience and Remote Sensing*, *39*(7), 1491–1507. https://doi.org/10.1109/36.934080

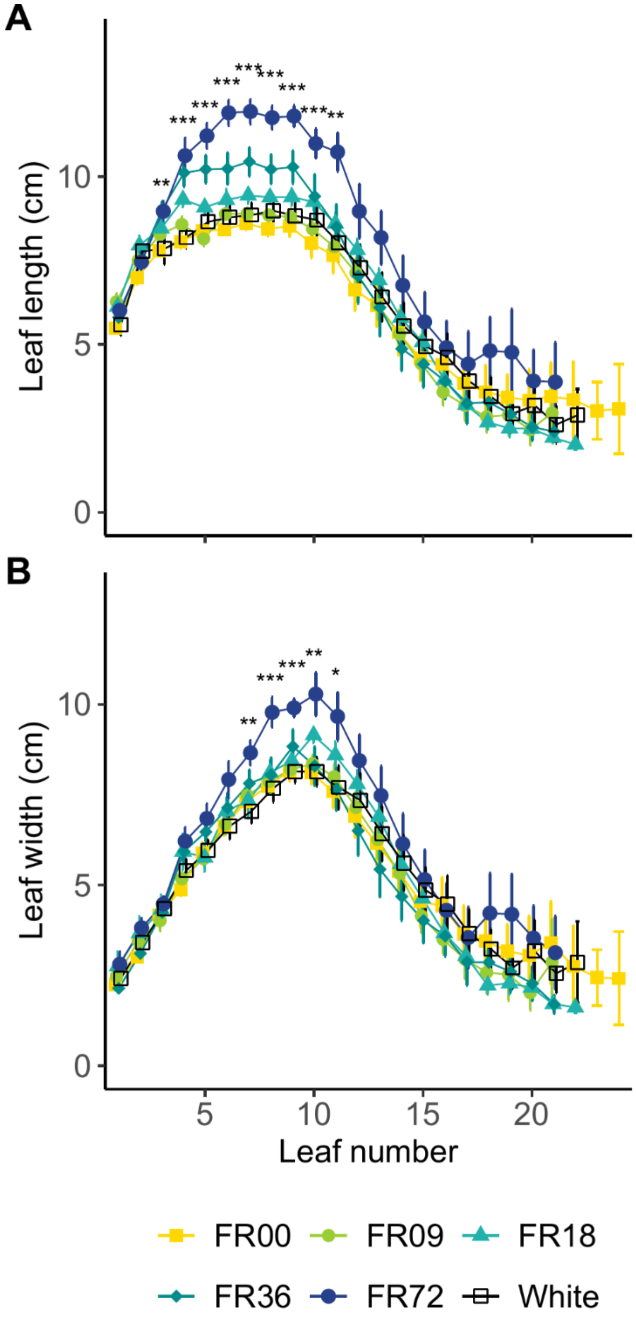

Supplementary Figure 2 Effect of far-red addition on **(A)** lettuce leaf length and **(B)** leaf width in function of leaf development, low numbers corresponding to the oldest true leaves, measured 14 DAT. Values shown are means ± SD, n ≥ 3. Different symbols mark significant differences between light quality treatments based on a Kruskal-Wallis test, * indicates significance at $P < 0.05$, ** indicates significance at $P < 0.01$ and *** indicates significance at $P < 0.001$. If no letters are displayed, no statistical differences were found based on the Kruskal-Wallis test.

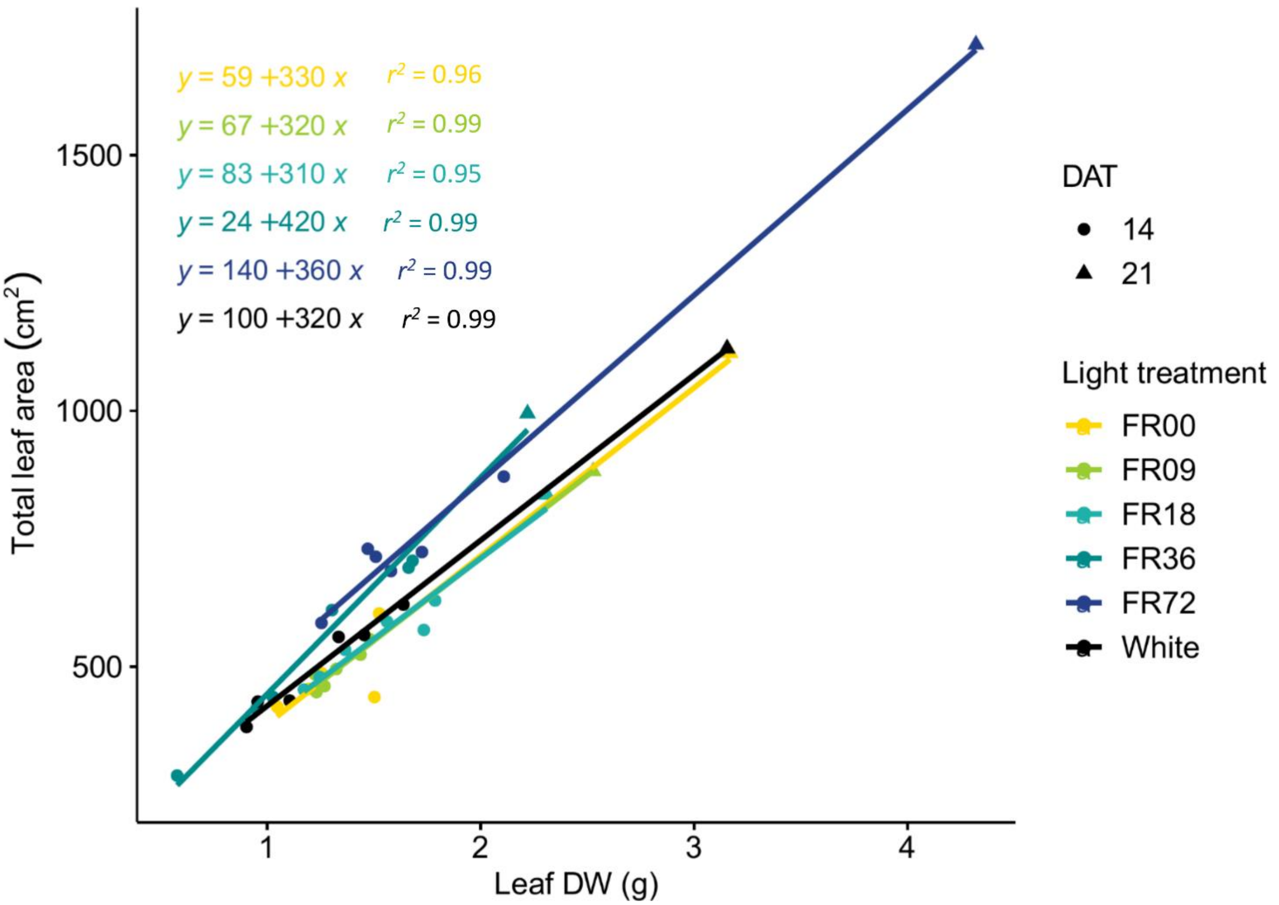

Supplementary Figure 3 Total leaf area of lettuce plants in function of total leaf dry weight, measured 14 and 21 DAT.

Supplementary Table 2 Effect of far-red addition on the selected vegetation indices. Data shown are means ± SE, n = 6. Different symbols mark significant differences between light quality treatments based on a One-Way ANOVA, * indicates significance at $P < 0.05$, ** indicates significance at $P < 0.01$ and *** indicates significance at $P < 0.001$. Different letters indicate statistical differences between light quality treatments based on Tukey's HSD ($P = 0.05$).

Light treatment code	R/FR	PSS	Far-red fraction	NDVI		G		Ctr1		GM1		CRI1	
FR00	∞	0.886	0.00	0.69 ± 0.01	a	3.76 ± 0.14	a	1.68 ± 0.04	b	2.44 ± 0.09	a	5.92 ± 0.36	a
FR09	29.29	0.874	0.04	0.67 ±0.01	ab	3.80 ± 0.22	a	1.71 ± 0.08	b	2.34 ± 0.06	ab	5.09 ± 0.39	ab
FR18	15.61	0.863	0.07	0.69 ± 0.01	a	4.07 ± 0.15	a	1.92 ± 0.07	ab	2.40 ± 0.10	a	5.90 ± 0.25	a
FR36	8.12	0.844	0.12	0.63 ± 0.01	bc	4.04 ± 0.13	a	2.05 ± 0.06	a	2.09 ± 0.03	bc	4.58 ± 0.28	b
FR72	4.27	0.809	0.21	0.61 ± 0.01	c	4.18 ± 0.06	a	2.12 ± 0.07	a	2.01 ± 0.02	c	4.38 ± 0.25	b
White	4.62	0.828	0.18	0.65 ± 0.01	abc	3.64 ± 0.09	a	1.70 ± 0.04	b	2.24 ± 0.04	abc	4.43 ± 0.20	b
P-value				< 0.001	***	0.08		< 0.001	***	< 0.001	***	< 0.001	***

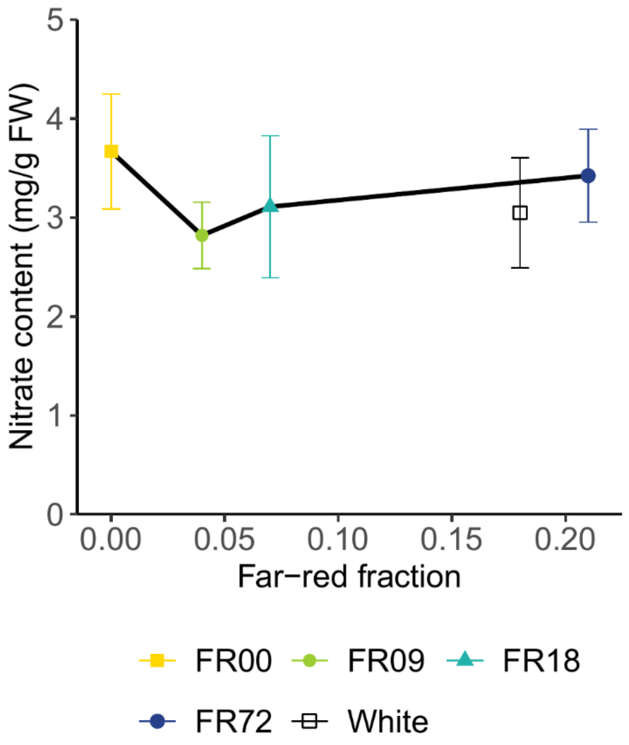

Supplementary Figure 4 Effect of far-red fraction on leaf nitrate content of lettuce cv. Alyssa, harvested 21 DAT. Values shown are means ± SE, n = 6. No statistical differences were found.
